# Patterns in hospital readmissions after ischaemic stroke – An observational study from the Swedish stroke register (Riksstroke)

**DOI:** 10.1177/2396987320925205

**Published:** 2020-06-15

**Authors:** Stefan Sennfält, Jesper Petersson, Teresa Ullberg, Bo Norrving

**Affiliations:** 1 Stroke Policy and Quality Register Research Group, Lund university, Sweden; 2 Department of Neurology, Skåne University Hospital, Lund, Sweden

**Keywords:** Ischaemic stroke, readmission, comorbidity

## Abstract

**Introduction:**

While acute treatment and secondary prevention in stroke have undergone major improvements, hospital readmission after index stroke remains high. However, there are few reports on long-term readmission patterns.

**Patients and methods:**

For this prospective observational study, data on demographics, functional status and living conditions were obtained from the Swedish Stroke Register (Riksstroke). Data on comorbidity and hospital readmissions up to five years post-index stroke were obtained from the Swedish National Patient Register. Patients were grouped based on number of readmissions: low (0–1) intermediate (2–4), high (5–9) or very high (≥10).

**Results:**

Of the 10,092 patients included, 43.7% had been readmitted within 12 months and 74.0% within 5 years. There was an average of three readmissions per individual during the five-year interval. A small group of patients with a high-comorbidity burden accounted for the majority of readmissions: approximately 20% of patients accounted for 60% of readmissions, and 5% of patients accounted for 25%. Circulatory conditions were the most common cause followed by infectious disease, stroke, trauma and diseases of the nervous system other than stroke. The proportion of readmissions due to stroke decreased sharply in the first six months.

**Conclusion:**

A small number of patients with a high degree of comorbidity accounted for the majority of hospital readmissions after index stroke. Our results highlight the need for further development of strategies to support high-risk comorbid stroke patients in the community setting. Further research describing characteristics and healthcare utilisation patterns in this group is warranted.

## Introduction

While acute treatment and secondary prevention in stroke have undergone major improvements over the last few decades, hospital readmission remains high. All-cause readmission has been reported in 39%–49% of patients within one year after index stroke^1–3^ and in 68%–83% of patients within five years.^2–4^ This compares to readmission rates in a similar-aged non-stroke population of 20% and 63% in one and five years, respectively.^2^ In stroke survivors, cardiac disease, recurrent stroke, infection and falls are cited as common causes for readmission, with recurrent stroke being particularly common in the first months.^1–6^

Stroke-related complications such as pneumonia, venous thromboembolism, dysphagia, incontinence, depression and cardiac complications are common^7^ and contribute to the high number of readmissions, particularly in the short term after stroke.^3^ In addition, several factors increase the physiological vulnerability of stroke patients, e.g. functional deterioration ensuing from the brain damage itself (stroke being the second leading cause of disability worldwide^8^), advanced age and a high-comorbidity burden.^9,10^

The high number of readmissions puts a strain on hospital-based healthcare.^11,12^ However, a significant proportion of these might be unnecessary^13^ and may actually be detrimental to vulnerable elderly individuals.^14^

The aim of the present study was to provide a comprehensive description of hospital readmission patterns in the first five years following ischaemic stroke (IS), and to characterise groups of patients based on readmission burden. We present data on demographics, comorbidity, readmission rate, predictive factors and causes of readmission.

## Patients and methods

### Study population

Approximately half of all patients registered in Riksstroke during 2011 were randomly selected for long-term follow-up.^15^ We excluded patients with intracerebral hemorrhage (ICH) or unspecified stroke (*n* = 1850), those under 18 years of age (*n* = 2), those of unknown functional status prior to stroke (*n* = 288), and those who died during index stroke hospital stay (*n* = 1000). In all, 10 092 individuals were included.

### Data

Riksstroke is the Swedish quality register for stroke care and has an estimated coverage of >90% of stroke patients admitted to hospital.^16,17^ The register includes data collected during admission, as well as survey data from paper-based follow-up questionnaires distributed to all registered patients at 3- and 12-month post-stroke (and additionally at three or five years for selected cohorts). Data on demographics, functional status and living conditions were obtained from the Riksstroke register. There were missing data in less than 2% of cases in baseline variables. There were missing data in 30.7% of cases for living conditions at 12 months and in 30.2% for functional status at 12 months.

Data on mortality status and date of death were obtained from the Swedish Causes of Death register.

Data on comorbidity were obtained from the Swedish National Patient Register (SNPR), which collects data on outpatient and inpatient healthcare contacts. Additional cases of dementia were identified through the Swedish Prescribed Drugs Register (SPDR). Data on a few conditions were obtained from the Riksstroke register (Supplementary Table I). The methodology was described in more detail in a previous paper.^10^

Data on primary diagnosis and date of admission were obtained from the SNPR for all readmissions from three weeks after index stroke (in 2011) until the end of 2016. Primary diagnoses were classified and grouped according to the International Statistical Classification of Diseases and Related Health Problems (ICD-10). In some cases, the original ICD-10 classification codes were merged or reclassified (Supplementary Table II).

Data on causes of hospital admission in the Swedish general population in 2018 were obtained from the online database of the Swedish National Board of Health and Welfare.

Data on the highest level of education were obtained from Statistics Sweden.

The study followed the Strengthening the Reporting of Observational Studies in Epidemiology (STROBE) statements.^18^

The local ethics approval committee (Regional Ethical Review Board, Lund) approved the project in 2017 (Dnr 2017/529). The committee waived the need for patient consent. Requests to access the dataset may be sent to Riksstroke after obtaining the appropriate ethics approval.

### Measures and definitions

We used the term readmission to include all admissions from day 22 to 1825 (five years) post-index stroke, i.e. not only the first but also all subsequent admissions.

The ICD-10 classification scheme allows for stroke to be assigned as the primary diagnosis if the reason for admission (within one year) is related to the index stroke. These cases might then be misinterpreted as recurrent stroke. Therefore, we consistently use the term ‘stroke’ rather than ‘recurrent stroke’ in the presentation of our results.

Readmission rate was reported per live person-years and was calculated by dividing the total number of readmissions during each three-month period by the number of patients alive at the beginning of this interval. The quotient was then multiplied by four.

Comorbidity burden was defined as the sum of individual comorbidities at the time of index stroke and categorised as none (0), low (1), moderate (2–3) and high (≥4). We included 17 specific chronic conditions that were selected with help from the Charlson Comorbidity Index (CCI)^19^ (Supplementary Table I).

Functional status was described using the modified Rankin Scale (mRS). We used information on dependency in several ADL domains (toileting, dressing, mobility), living conditions, and need of support from next of kin using a validated and previously specified translation algorithm.^20^ Independency was defined as mRS ≤2 without home care service.

Level of consciousness at admission (registered using the Reactions Level Scale 85 [RLS] ^21^) was used as a proxy for stroke severity: alert (RLS 1), drowsy (RLS 2–3) and comatose (RLS 4–8).

Highest level of education was used as a proxy for socioeconomic status.

Patients alive 12 months post-index stroke (*n* = 8450) were grouped based on cumulative number of hospital readmissions during the follow-up period: low (0–1), intermediate (2–4), high (5–9) and very high (≥10).

### Statistical methods

All statistical analyses were conducted using IBM SPSS version 24.

Categorical variables were summarised as proportions (percentages), and quantitative variables (age only) as medians.

Kaplan–Meier curves were used to calculate probability of readmission (1-survival) within specific time intervals. Patients were censored upon death. Analyses were stratified based on comorbidity burden at index stroke and living conditions at three-month post-index stroke. The log-rank test was used to test for significance. For the stratified analysis based on living conditions at three months, only patients alive at three months were included (*n* = 9370). Patients in assisted living were divided into two groups based on whether or not they were in assisted living before index stroke.

Cox-proportional hazards’ regression models were used to calculate hazard ratios (HR) with 95% confidence intervals (CI) for predictors of readmission within 12 months and 5 years. Previous stroke and dementia were included as separate variables and excluded from the variable ‘total comorbidity burden’.

## Results

### Patient characteristics

We included 10,092 patients. Median age was 78 years, 52.7% were male and 49.5% had secondary education or higher (Table 1). The majority, 82.0%, displayed some degree of comorbidity at the time of index stroke and 19.2% had a high comorbidity burden. The most common comorbidities were hypertension (62.0%), atrial fibrillation (28.4%), diabetes (20.8%), previous stroke (22.2%) and non-metastatic solid tumor (11.7%). The proportion of patients who were functionally independent pre-index stroke was 73.1%, and 7.9% were in assisted living.

**Table 1.** Patient characteristics, all included patients.

	*n* = 10,092
Demographics	
Age (median [IQR])	78 (17)
Male sex	5323 (52.7)
Female sex	4769 (47.3)
Highest level of education	
Primary	5022 (50.6)
Secondary	3504 (35.3)
≥Tertiary	1399 (14.1)
Pre-stroke functional status	
mRS 0–2 (independent)	7378 (73.1)
mRS 3	1612 (16.0)
mRS 4	794 (7.9)
mRS 5	308 (3.1)
Living conditions before stroke	
Own home	7622 (75.5)
Own home with home care service	1623 (16.1)
Assisted living	798 (7.9)
Other	49 (0.5)
Total comorbidity burden	
None	1818 (18.0)
Low	2557 (25.3)
Moderate	3777 (37.4)
High	1940 (19.2)
Selected comorbidities	
Angina pectoris	999 (9.9)
Atrial fibrillation	2868 (28.4)
Chronic kidney failure	275 (2.7)
COPD	404 (4.0)
Congestive heart failure	1051 (10.4)
Dementia	423 (4.2)
Diabetes	2101 (20.8)
Hypertension	6253 (62.0)
Myocardial infarction	585 (5.8)
Previous stroke	2242 (22.2)
Solid tumor, non-metastatic	1178 (11.7)

Presented as no. (%) unless otherwise stated.IQR: interquartile range; COPD: chronic obstructive pulmonary disease; mRS: modified Rankin scale.

### Readmission rate and predictive factors

**Figure 1.**
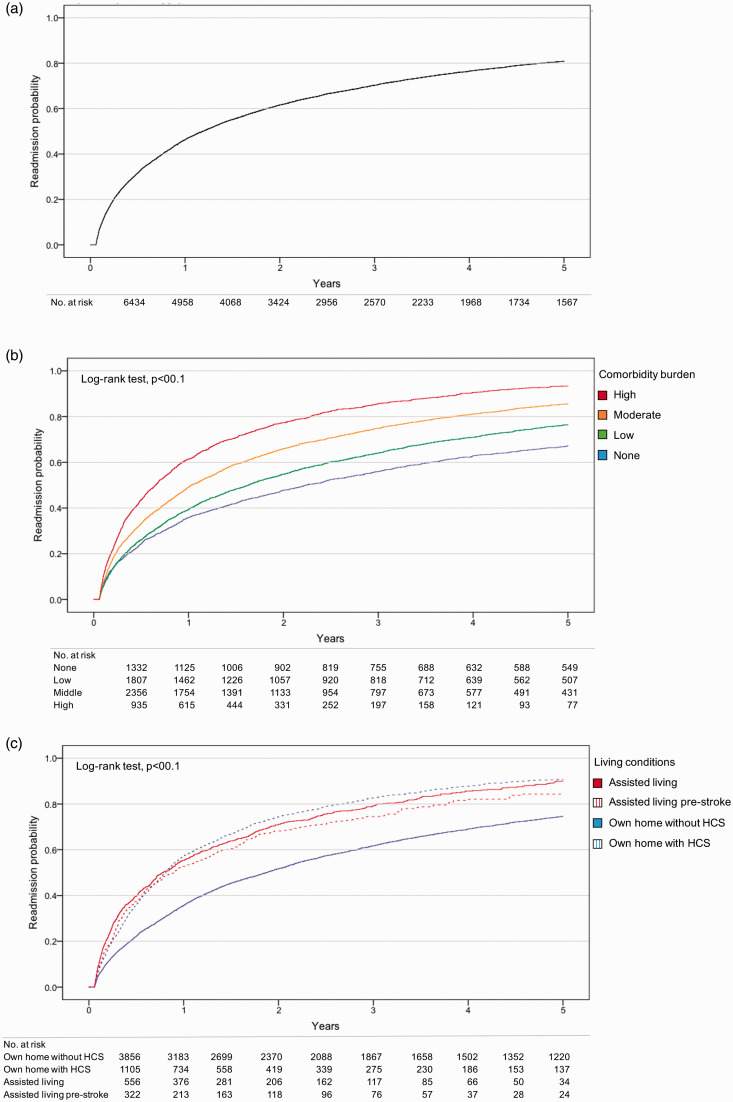
Probability of readmission. (a) In all included patients, (b) stratification based on comorbidity burden and (c) stratification based on living conditions at three months. HCS: home care service.

The probability of readmission in all patients was 0.46 within 12 months and 0.81 within 5-years post-index stroke (Figure 1(a)). The proportion of patients that had been readmitted was 43.7% at 12 months and 74.0% at 5 years. An additional 7.2% and 10.5% of patients had died within 12 months and 5 years, respectively, without being readmitted (Supplementary Table III).

When stratifying by comorbidity burden, the probability curves diverged substantially (*P* < 001; Figure 1(b)). The probability of readmission within 12 months for none, low-, moderate- and high-comorbidity burden was 0.36, 0.39, 0.49 and 0.61, respectively. In addition, stratification based on living conditions at three months revealed a significantly lower probability of readmission for patients living in their own home *without* home care service compared to other groups (*P* < 001; Figure 1(c)).

**Figure 2.**
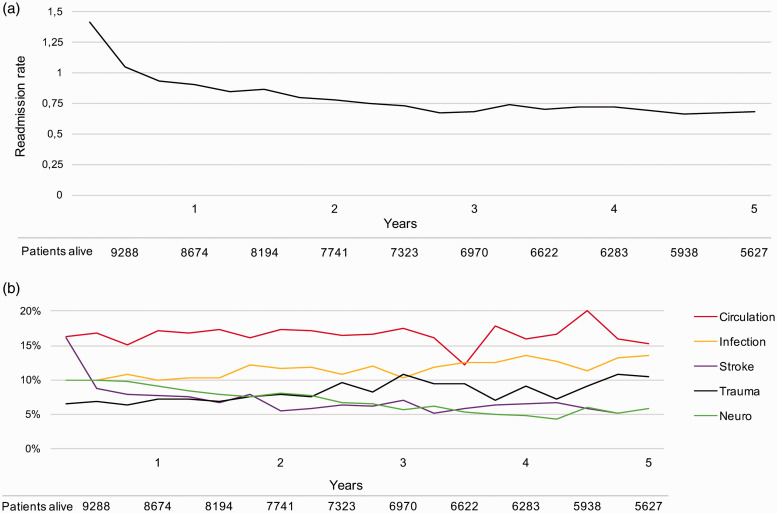
Readmission rate and causes of readmission over time. (a) Readmission rate per live person-years during each consecutive Three-month period. (b) Proportions of the five most common causes of readmission (diagnostic groups) over time. Neuro: diseases of the nervous system other than stroke.

Readmission rate varied over time: 1.42 per live person-years between one and four months, declining during the first year to stabilise at 0.66–0.87 throughout the remaining follow-up period (Figure 2(a)).

The strongest predictive factor for readmission within 12 months or 5 years was total comorbidity burden: 12-month HR was 1.75 (95% CI 1.57–1.95) and 5-year HR was 1.85 (95% CI 1.70–2.01) for high comorbidity compared to no comorbidity (Table 2). Previous stroke was independently associated with a slightly increased probability of readmission, whereas dementia was associated with a decreased probability.

**Table 2.** Predictors of readmission within 12 months and 5 years.

	12 months HR (95% CI)	5 years HR (95% CI)
Female sex	1.03 (0.97–1.10)	1.00 (0.95–1.05)
Age	1.002 (0.999–1.005)	1.010 (1.008–1.012)
Previous stroke	1.18 (1.10–1.27)	1.18 (1.11–1.24)
Dementia	0.86 (0.73–1.01)	0.85 (0.74–0.97)
Highest level of education		
Primary (reference)	–	–
Secondary	1.04 (0.97–1.12)	1.02 (0.96–1.07)
≥Tertiary	0.97 (0.89–1.07)	0.97 (0.91–1.04)
Pre-stroke functional status		
mRS 0–2 (reference)	–	–
mRS 3	1.33 (1.22–1.45)	1.28 (1.20–1.38)
mRS 4	1.22 (1.08–1.38)	1.20 (1.08–1.32)
mRS 5	1.17 (0.96–1.44)	1.16 (0.97–1.38)
Level of consciousness at admission		
Alert (reference)	–	–
Drowsy	1.24 (1.11–1.39)	1.18 (1.08–1.30)
Comatose	1.59 (1.26–2.01)	1.43 (1.16–1.76)
Total comorbidity burden		
None (reference)	–	–
Low	1.09 (1.00–1.20)	1.17 (1.09–1.26)
Moderate	1.34 (1.22–1.46)	1.42 (1.33–1.52)
High	1.75 (1.57–1.95)	1.85 (1.70–2.01)
Discharge destination		
Independent living (reference)	–	–
Assisted living	1.12 (1.03–1.23)	1.06 (0.98–1.14)
Rehabilitation/geriatric unit/other	1.40 (1.28–1.52)	1.26 (1.18–1.35)

*n* = 9612.

CI: confidence interval; HR: hazard ratio; mRS: modified Rankin scale.

### Distribution of number of readmissions

**Figure 3.**
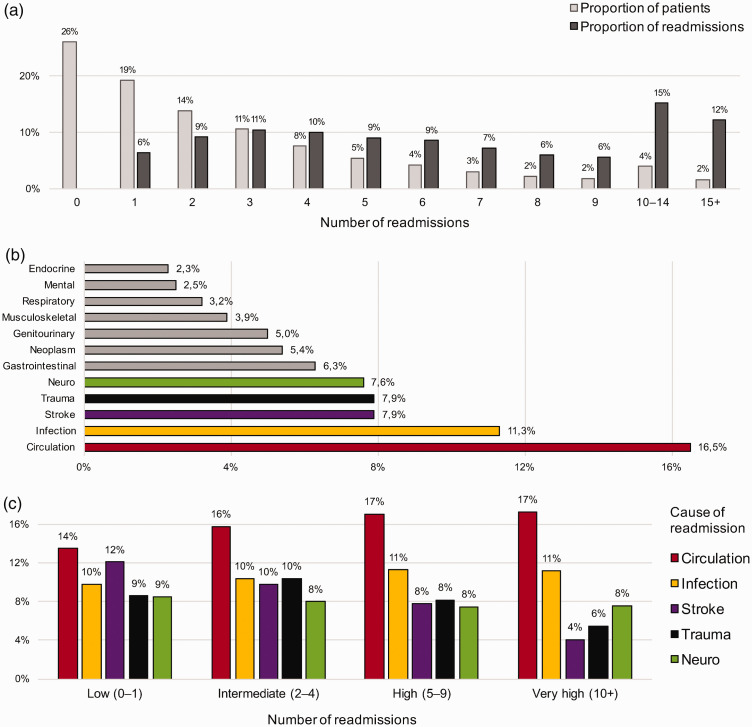
Distribution of causes of readmission. (a) Proportion of patients with *x* number of readmissions and the proportion of the total number of readmission accounted for by these patients. For instance, 19% of patients had one readmission which accounted for 6% of the total number of readmissions. (b) Proportions of causes of readmission (diagnostic groups) for all patients. Miscellaneous (20.2%) not shown. (c) Proportions of the five most common causes of readmission (diagnostic groups) in patients with different number of readmissions, alive at 12 months. *n* = 8450. Neuro: diseases of the nervous system other than stroke.

The total number of readmissions was 9660 (0.96 per patient) within 12 months and 30 454 (3.02 per patient) within 5 years. However, these were unevenly distributed between individuals: 45.2% had 0–1 readmissions, which only accounted for 6.4% of the total number, while 22.7% of patients had ≥5 readmissions, which accounted for 63.8% of the total number, and small group (5.7%) had ≥10 readmissions, which accounted for 27.4% (Figure 3(a)).

Patients alive at 12 months were grouped based on number of readmissions: low (0–1, 40.8%), intermediate (2–4, 33.2%), high (5–9, 19.2%) and very high (≥10, 6.8%) (Table 3). Median age was significantly lower (*P* < 001) in those with a low number of readmissions (73 years) or very high number of readmissions (74 years) compared to those with an intermediate or high number (78 years).

**Table 3.** Patient characteristics, stratified based on number of readmissions.

	Low *n* = 3449	Intermediate *n* =2806	High *n* = 1624	Very high *n* = 571	Total *n* = 8450
Demographics					
Age (median [IQR])	73 (18)	78 (16)	78 (13)	74 (14)	76 (16)
Male sex	1876 (54.4)	1489 (53.1)	895 (55.1)	318 (55.7)	4578 (54.2)
Level of consciousness at admission					
Alert	3225 (94.3)	2558 (92.2)	1484 (92.8)	509 (90.6)	7776 (93.0)
Drowsy	172 (5.0)	187 (6.7)	102 (6.4)	46 (8.2)	507 (6.1)
Comatose	24 (0.7)	29 (1.0)	14 (0.9)	7 (1.2)	74 (0.9)
Functional status at 12 months					
mRS 0–2 (independent)	1789 (71.6)	1051 (53.7)	518 (47.4)	175 (50.7)	3533 (59.9)
mRS 3	298 (11.9)	409 (20.9)	279 (25.5)	82 (23.8)	1068 (18.1)
mRS 4	209 (8.4)	333 (17.0)	232 (21.2)	76 (22.0)	850 (14.4)
mRS 5	202 (8.1)	165 (8.4)	64 (5.9)	12 (3.5)	443 (7.5)
In assisted living at 12 months	281 (11.1)	295 (14.9)	124 (11.2)	24 (6.7)	724 (12.1)
Total comorbidity burden					
None	950 (27.5)	473 (16.9)	206 (12.7)	62 (10.9)	1691 (20.0)
Low	1042 (30.2)	786 (28.0)	352 (21.7)	89 (15.6)	2269 (26.9)
Moderate	1094 (31.7)	1109 (39.5)	709 (43.7)	232 (40.6)	3144 (37.2)
High	363 (10.5)	438 (15.6)	357 (22.0)	188 (32.9)	1346 (15.9)
Selected comorbidities					
Atrial fibrillation	726 (21.0)	743 (26.5)	478 (29.4)	182 (31.9)	2129 (25.2)
Chronic kidney failure	38 (1.1)	48 (1.7)	54 (3.3)	41 (7.2)	181 (2.1)
COPD	61 (1.8)	105 (3.7)	94 (5.8)	53 (9.3)	313 (3.7)
Congestive heart failure	167 (4.8)	235 (8.4)	195 (12.0)	103 (18.0)	700 (8.3)
Dementia	130 (3.8)	87 (3.1)	37 (2.3)	10 (1.8)	264 (3.1)
Diabetes	579 (16.8)	524 (18.7)	403 (24.8)	190 (33.3)	1696 (20.1)
Hypertension	1884 (54.6)	1787 (63.7)	1091 (67.2)	390 (68.3)	5152 (61.0)
Myocardial infarction	107 (3.1)	146 (5.2)	114 (7.0)	66 (11.6)	433 (5.1)
Previous stroke	556 (16.2)	602 (21.6)	410 (25.5)	157 (27.8)	1725 (20.6)

Presented as no. (%) unless otherwise stated. Only patients alive at 12 months (*n* = 8450) were included.

COPD: chronic obstructive pulmonary disease; IQR: interquartile range; mRS: modified Rankin scale.

Low = 0–1 condition, intermediate = 2–4, high = 5–9, very high ≥10.

The proportion of high comorbidity burden (≥4 conditions) increased from 10.5% in those with a low number of readmissions to 32.9% in those with a very high number. Individual comorbidities with the largest relative increases were chronic kidney failure (1.1%–7.2%), chronic obstructive pulmonary disease (1.8%–9.3%), myocardial infarction (3.1%–11.6%) and congestive heart failure (4.8%–18.0%).

### Causes of readmission

The five most common causes of readmission were, in descending order: circulatory conditions (16.5%), infectious diseases (11.3%), stroke (7.9%), trauma (7.9%) and diseases of the nervous system other than stroke (7.6%) (Figure 3(b)). A detailed description is provided in Supplementary Table II. The relative proportions varied over time (Figure 2(b)). The proportion of readmissions due to stroke decreased sharply during the first six-month post-index stroke from 16.1% to 8.0%. There was an increasing trend in readmission due to infectious disease and trauma, while the proportion of readmissions due to diseases of the nervous system other than stroke decreased.

The relative proportions of causes of readmission varied in groups of different number of readmissions (Figure 3(c)). The proportion of readmissions due to stroke was 12.1% in patients with a low number of readmissions compared to 4.1% in those with a very high number. Similarly, the proportion of readmissions due to trauma was 8.6% in patients with a low number of readmissions compared to 5.5% in those with a very high number. The proportions of readmissions due to circulatory conditions increased slightly with increasing number of readmissions.

## Discussion

Of the 10,092 patients included in the study, 43.7% had been readmitted within 12 months and 74.0% within 5 years. The readmission rate was highest in the first year post-index stroke, and circulatory conditions were the most common cause of readmission, followed by infectious disease and stroke. A small group of individuals accounted for the majority of readmissions: approximately 20% of patients accounted for 60% of readmissions and approximately 5% of patients accounted for 25%. Comorbidity burden was greater in those with a higher number of readmissions and the causes for readmission varied slightly.

### Our results in context

In the present study, 43.7% and 74.0% of patients had been readmitted within 12 months and 5 years, respectively. Comparable studies, predominantly including ischaemic stroke, report similar results: 40.3–40.4% readmission within 12 months and 67.5–68.0% within 5 years of index stroke.^4,5^ In a study only including ICH patients, readmission at 12 months was similar: 40.6%.^22^ However, there was a high pre-discharge mortality, approximately 25% of the original cohort, likely including many vulnerable individuals who might otherwise have contributed with readmissions. In the non-stroke elderly population, admissions are significantly less common; estimated to 20% and 63% in one and five years, respectively.^2^

The initially high, but rapidly decreasing rate of readmission described by us can also be seen in patients hospitalised for other conditions, e.g. myocardial infarction. For instance, Khot et al. reported an initial rate of 1.8 per person-year which decreased to less than 0.2 at 12 months and a small proportion (approximately 5%) accounted for almost half of readmissions.^23^

In our study, circulatory conditions were the most common cause for readmission throughout the follow-up period, whereas readmission due to stroke decreased sharply during the first six months. A similar overall pattern can be discerned in previous research.^1,3,24^ The admission pattern in the general population differs in a number of ways. The top five causes of hospital admission in the Swedish general population ≥65 years of age in 2018 were circulatory conditions (17.6%), trauma (11.4%), respiratory disease (10.4%), neoplasm (9.8%) and gastrointestinal disease (8.2%), while cerebrovascular disease only accounted for 3.9%.^25^ Thus, diseases of the respiratory and digestive systems are relatively more common reasons for admission in the general population as compared to stroke patients, whereas cerebrovascular disease is less common. Also, admission rates are substantially lower in the general population: 0.34 per year for individuals ≥65 years of age in 2018,^25^ compared to between 0.66 and 1.42 in stroke patients in our study over the five-year follow-up period.

### Implications of our results

With an ageing population and healthcare systems under an increasing amount of strain, efficient management strategies and resource allocation are crucial. We show that a small group of stroke patients with a large comorbidity burden account for the bulk of long-term readmissions and associated costs. These individuals represent an important target for community-based pre-emptive interventions to reduce morbidity, as many complications post-stroke could potentially be avoided.^26^

The high prevalence of comorbidity and stroke-related complications warrant a proactive, coordinated and structured approach. Strategies for structured post-stroke checklists and intervention programs are currently being developed.^27,28^ In addition, the clinical concept of frailty may be a useful tool to identify and assess high-risk individuals. Frailty is the result of cumulative decline in many physiological systems during a lifetime, leading to increased vulnerability to physiological stressors and consequently, increased risk of death and institutionalisation.^29^ It is common in the elderly and it has been suggested that frailty assessment should be integrated into medical care of all patients over 70.^30^ Ideally, this would be part of a continuous patient-centered follow-up program, possibly administered by primary care providers, who are naturally positioned to work as a unifying force in stroke management, forging strong long-term relationships with both patients and informal caregivers.

We show that individuals living at home with home care service at three months were at highest risk for readmission. They were likely more vulnerable (and more likely to require hospital admission) than those living at home without home care service, but at the same time they would not have had access to community-based health care and support to the same degree as those in assisted living.

Also, a group that merits particular attention is stroke survivors in assisted living. The decision whether or not to admit these individuals is complicated and involves considering potential physical and mental deterioration that can be brought on by hospitalisation.^14^ In many cases, it is questionable whether hospital admission is warranted or if adequate care could be received in the assisted living setting through primary care. Previous research suggests that as many as 40% of admissions from nursing homes might be inappropriate.^31^ In our study, discharge to assisted living showed a small but significant association with a greater probability of readmission within 12 months. The topic of hospital admission in stroke patients in assisted living needs to be explored further.

### Strengths

The large patient sample, long follow-up period and comprehensive approach are major strengths. Also, whereas previous research rarely presents stratified results, our analyses revealed important differences between patients with different numbers of readmissions.

### Limitations

First, the Riksstroke register only includes stroke cases admitted to hospital, which means that cases of minor stroke and patients solely managed in primary care may be missing from our dataset.

Second, the SNPR does not include data from primary care, which may have under-estimated the comorbidity burden, especially for conditions not requiring specialist care.

Third, when selecting for high number of readmissions, there was also an indirect selection for longevity. To remedy this problem, we only included patients who were alive at the 12-month follow-up for the comparative analyses of groups of patients with different numbers of readmissions.

Forth, a major issue when comparing results to previous research is the considerable variability in definitions, inclusion criteria, analytic approaches and follow-up periods.

Last, since both readmission in the first year due to circumstances related to index stroke and readmission due to recurrent stroke are registered using the same ICD-10 code, it is not possible to differentiate between the two. This limits the conclusions that can be drawn from our results since these two causes for readmission are fundamentally different.

## Conclusion

Within 12 months post-index stroke, 43.7% of patients had been readmitted, with 74.0% readmitted within 5 years. There was an average of three hospital readmissions per patient, but a small group accounted for the majority of readmissions: approximately 20% of patients were responsible for 60% of readmissions and 5% of patients for 25%. Patients with a higher number of readmissions had a greater degree of comorbidity and displayed a somewhat different pattern of readmission causes. Our results highlight the need for further development of strategies to support high-risk comorbid stroke patients in the community setting. Further research describing characteristics and health-care utilisation patterns in this group is warranted.

## Supplemental Material

sj-pdf-1-eso-10.1177_2396987320925205 - Supplemental material for Patterns in hospital readmissions after ischaemic stroke – An observational study from the Swedish stroke register (Riksstroke) Click here for additional data file. Supplemental material, sj-pdf-1-eso-10.1177_2396987320925205 for Patterns in hospital readmissions after ischaemic stroke – An observational study from the Swedish stroke register (Riksstroke) by Stefan Sennfält, Jesper Petersson, Teresa Ullberg and Bo Norrving in European Stroke Journal
