# Supplementary material for: Patterns in hospital readmissions after ischaemic stroke – An observational study from the Swedish stroke register (Riksstroke)
Source: Eur Stroke J. 2020 Jun 15;5(3):286–96. doi: 10.1177/2396987320925205 (PMC7538769; doi:10.1177/2396987320925205)
Supplement: sj-pdf-1-eso-10.1177_2396987320925205 - Supplemental material for Patterns in hospital readmissions after ischaemic stroke – An observational study from the Swedish stroke register (Riksstroke) [file sj-pdf-1-eso-10.1177_2396987320925205.pdf]

# Supplementary material

**Supplementary table I.** List of the 17 included conditions and ICD-10 codes.

| | **ICD-10 code** |
|---|---|
| **CCI conditions included** | |
| Solid tumor, non-metastatic | C00–76 |
| Solid tumor, metastatic | C77–79 |
| Leukemia/myeloma | C88–96 |
| Lymphoma | C81–86 |
| Chronic liver disease | B18, K70, K72, K73, K74 |
| Chronic kidney failure | N18 |
| COPD | J44 |
| Rheumatoid arthritis | M04, M05 |
| Peripheral vascular disease | I73 |
| Congestive heart failure | I50 |
| Myocardial infarction | I21, I22 |
| Diabetes | Data obtained from Riksstroke |
| Dementia | F00–03 |
| Previous stroke | Data obtained from Riksstroke |
| **CCI conditions not included** | |
| Ulcer disease | - |
| Hemiplegia | - |
| AIDS | - |
| **Non-CCI conditions included** | |
| Atrial fibrillation/flutter | Data obtained from Riksstroke |
| Angina pectoris | I20 |
| Hypertension | Data obtained from Riksstroke |

CCI=Charlson comorbidity index, COPD=chronic obstructive pulmonary disease.

**Supplementary table II.** Primary diagnosis of each readmission, presented as diagnostic groups.

| | n=30 287 |
|---|---|
| **Infectious** | **3414 (11.3)** |
| Intestinal | 237 (0.8) |
| Sepsis | 345 (1.1) |
| Skin infection, erysipelas and mycosis | 264 (0.9) |
| Acute upper respiratory and influenza | 213 (0.7) |
| Pneumonia and acute bronchitis | 1609 (5.3) |
| Cystitis and pyelonephritis | 326 (1.1) |
| Miscellaneous | 420 (1.4) |
| **Neoplasm** | **1647 (5.4)** |
| Malignant solid neoplasm | 1201 (4.0) |
| Leukemia/lymphoma | 313 (1.0) |
| Benign neoplasm | 133 (0.4) |
| **Endocrine/nutritional and metabolic** | **695 (2.3)** |
| Diabetes mellitus | 361 (1.2) |
| Nutritional/metabolic disease | 272 (0.9) |
| Miscellaneous | 62 (0.2) |
| **Mental and behavioral disorders** | **757 (2.5)** |
| Mood disorders | 160 (0.5) |
| Mental and behavioral disorders due to use of alcohol | 192 (0.6) |
| Miscellaneous | 405 (1.3) |
| **Nervous system other than stroke** | **3309 (7.6)** |
| Dementia | 179 (0.6) |
| Epilepsy | 670 2.2) |
| TIA and related syndromes | 600 (2.0) |
| Paresis | 82 (0.3) |
| Sequelae of CVD | 473 (1.6) |
| Miscellaneous | 305 (1.0) |
| **Stroke** | **2392 (7.9)** |
| Stroke: ICH | 288 (0.9) |
| Stroke: IS | 2104 (6.9) |
| **Diseases of the eye** | **105 (0.3)** |
| **Diseases of the ear** | **125 (0.4)** |
| **Circulatory** | **5007 (16.5)** |
| Ischemic heart disease | 1196 (3.9) |
| Atrial fibrillation and other arrhythmias | 923 (3.0) |
| Heart failure | 1242 (4.1) |
| PE/Venous thrombosis | 186 (0.6) |
| PVD | 561 (1.9) |
| Hypertension | 147 (0.5) |
| Hypotension | 183 (0.6) |
| Miscellaneous | 569 (1.9) |
| **Gastrointestinal** | **1922 (6.3)** |
| Peptic ulcer | 131 (0.4) |
| Non infective enteritis and colitis | 451 (1.5) |

| | |
|---|---|
| Hernia | 133 (0.4) |
| Paralytic Ileus | 146 (0.4) |
| Gallbladder and biliary tract | 354 (1.2) |
| GI bleed | 306 (1.0) |
| Miscellaneous | 401 (1.3) |
| **Skin other than infection** | **135 (0.4)** |
| **Respiratory** | **970 (3.2)** |
| COPD, emphysema and chronic bronchitis | 459 (1.5) |
| Respiratory insufficiency | 88 (0.3) |
| Miscellaneous | 423 (1.4) |
| **Musculoskeletal** | **1208 (3.9)** |
| Rheumatoid arthritis | 41 (0.1) |
| Osteoarthritis | 229 (0.8) |
| Back pain (dorsalis) | 135 (0.4) |
| Spondylopathies | 219 (0.7) |
| Miscellaneous | 584 (1.9) |
| **Genitourinary** | **1525 (5.0)** |
| Urolithiasis | 104 (0.3) |
| Chronic kidney failure | 226 (0.7) |
| Acute kidney failure | 79 (0.3) |
| Miscellaneous | 1116 (3.7) |
| **Congenital malformations** | **28 (0.1)** |
| **Symptoms and findings not elsewhere specified** | **4182 (13.8)** |
| Pain in throat and chest | 658 (2.2) |
| Abdominal and pelvic pain | 298 (1.0) |
| Nausea and vomiting | 78 (0.3) |
| Hematuria | 166 (0.5) |
| Dysphagia | 57 (0.2) |
| Vertigo | 497 (1.6) |
| Headache | 109 (0.4) |
| Malaise and fatigue | 164 (0.5) |
| Syncope | 379 (1.2) |
| Convulsions | 221 (0.7) |
| Anemia | 368 (1.2) |
| Miscellaneous | 1187 (3.9) |
| **Injury of external cause (Trauma)** | **2390 (7.9)** |
| Hip fracture | 846 (2.8) |
| Other fracture | 630 (210) |
| Other injury | 731 (2.4) |
| Miscellaneous | 183 (0.6) |
| **Complication medical of surgical care** | **452 (1.5)** |
| **Factors influencing health status and contact with health services** | **1011 (3.3)** |

From day 22 to 1825 (five years) after index stroke.
Presented as No. (%).
COPD=chronic obstructive pulmonary disease,
CVD=cerebrovascular disease, ICH=intracerebral hemorrhage,
IS=ischemic stroke, PVD=peripheral vascular disease, TIA=transient ischemic attack

**Supplementary table III.** Proportions of patients readmitted or deceased at different time points after index stroke.

| | Admitted | | | Not admitted | | | Readmitted or dead |
|---|---|---|---|---|---|---|---|
| | Total | Alive | Dead | Total | Alive | Dead | |
| 6 months | 3031 (30.0) | 2562 (25.4) | 469 (4.6) | 7061 (70.0) | 6447 (63.9) | 614 (6.1) | 3645 (36.1) |
| 12 months | 4411 (43.7) | 3491 (34.6) | 920 (9.1) | 5681 (56.3) | 4959 (49.1) | 722 (7.2) | 5133 (50.9) |
| 3 years | 6569 (65.1) | 4232 (41.9) | 2337 (23.2) | 3523 (34.9) | 2571 (25.5) | 952 (9.4) | 7521 (74.5) |
| 5 years | 7464 (74.0) | 3907 (38.7) | 3557 (35.2) | 2628 (26.0) | 1567 (15.5) | 1061 (10.5) | 8525 (84.5) |

n=10 092. Presented as no. (%).
